# Cost-effectiveness analysis of malaria interventions using disability adjusted life years: a systematic review

**DOI:** 10.1186/s12962-017-0072-9

**Published:** 2017-07-01

**Authors:** Resign Gunda, Moses John Chimbari

**Affiliations:** School of Nursing and Public Health, College of Health Sciences, University of KwaZulu-Natal, Howard Campus, Durban, 4001 South Africa

**Keywords:** Malaria, Disability-adjusted life years, Cost-effectiveness, Cost, Effectiveness

## Abstract

**Background:**

Malaria continues to be a public health problem despite past and on-going control efforts. For sustenance of control efforts to achieve the malaria elimination goal, it is important that the most cost-effective interventions are employed. This paper reviews studies on cost-effectiveness of malaria interventions using disability-adjusted life years.

**Methods:**

A review of literature was conducted through a literature search of international peer-reviewed journals as well as grey literature. Searches were conducted through Medline (PubMed), EMBASE and Google Scholar search engines. The searches included articles published in English for the period from 1996 to 2016. The inclusion criteria for the study were type of malaria intervention, year of publication and cost-effectiveness ratio in terms of cost per DALY averted. We included 40 studies which specifically used the DALY metric in cost-effectiveness analysis (CEA) of malaria interventions.

**Results:**

The majority of the reviewed studies (75%) were done using data from African settings with the majority of the interventions (60.0%) targeting all age categories. Interventions included case treatment, prophylaxis, vector control, insecticide treated nets, early detection, environmental management, diagnosis and educational programmes. Sulfadoxine–pyrimethamine was the most common drug of choice in malaria prophylaxis, while artemisinin-based combination therapies were the most common drugs for case treatment. Based on guidelines for CEA, most interventions proved cost-effective in terms of cost per DALYs averted for each intervention.

**Conclusion:**

The DALY metric is a useful tool for determining the cost-effectiveness of malaria interventions. This paper demonstrates the importance of CEA in informing decisions made by policy makers.

## Background

Although the number of malaria cases are showing a declining trend, about 3.2 billion people remain at risk of malaria [1]. There were an estimated 214 million new cases of malaria and 438,000 deaths in 2015 alone, with approximately 80% of these deaths concentrated in just 15 countries, mainly in Africa [1]. The main challenges in the fight against malaria include in-effective national malaria control programmes, changes in population distribution and population growth, changes in land use, resistance to anti-malarial drugs, insecticide resistance, poor health infrastructure as well as climate change and climate variability [1, 2].

Cost-effectiveness analysis (CEA) of malaria interventions can provide essential information for malaria control at various levels and can inform health sector budgets [3]. In this context, intervention is defined as any preventive, promotive, curative, or rehabilitative action that improves health [4]. CEA uses a cost-effectiveness ratio (CER) to compare interventions in terms of costs and effectiveness [5]. CEA can be used to identify priority interventions when resources are limited [6] and hence should be considered when designing strategies for prevention, treatment and control of disease [7]. CEA takes into account the costs and effects of adding new interventions to current ones or of replacing an existing intervention with another targeting the same condition [4]. The decision to employ a particular malaria intervention must therefore be determined, not only by the effectiveness of the intervention, but also by the ability of the health system to sustain its use [8].

The disability-adjusted life year (DALY) is a metric measure for burden of disease [9, 10] developed by the World Health Organization (WHO), World Bank and the Harvard School of Public Health researchers [10–13]. DALYs were first used in the global burden of disease and injury (GBD) study, a joint study done by the World Bank, WHO and Harvard School of Public Health [10, 14]. The DALY can be used as a summary measure to determine the cost-effectiveness of different types of interventions for each specific disease type [15]. It has been recommended that analysts express CER in terms of DALYs, although measures such as the quality-adjusted life years (QALYs) and healthy life years (HYL) can also be used [15]. CERs can be expressed as the cost per DALY averted through each intervention thereby allowing for comparisons in costs and effectiveness across different settings.

Information on CEA of malaria interventions is essential in informing malaria control programmes to guide the decision-making and planning processes. There is paucity of information on CEA of malaria interventions in most low- and medium-income countries, which are home to the majority of the impoverished communities of the world [15]. This may result in failure to effectively implement intervention programs at sufficient scale [5]. Hence, this review was conducted to assess and examine the utility of the DALY methodology in CEA of malaria interventions.

## Methods

**Fig. 1:**
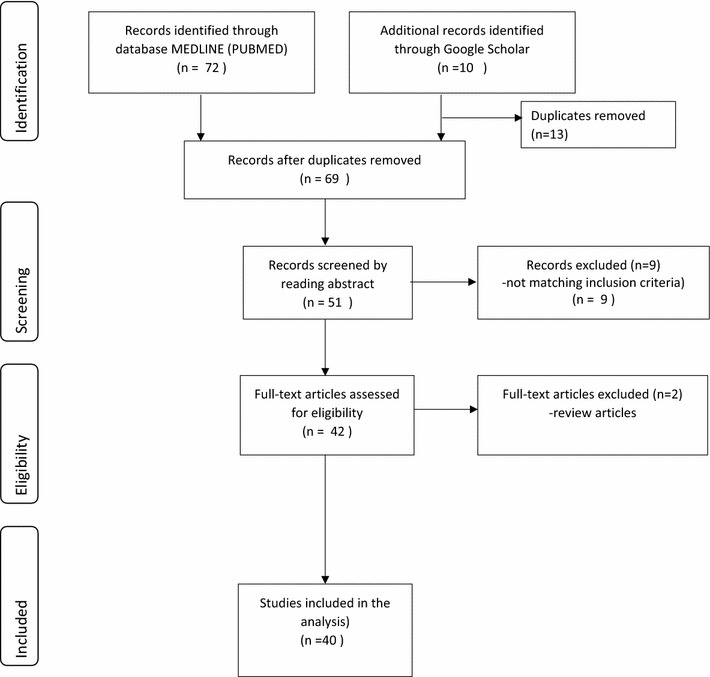
PRISMA flow diagram

In this review, only studies employing the DALY methodology in CEA of malaria interventions were included. Selection for eligible studies was conducted through a search of peer-reviewed journals on Medline (PubMed) and EMBASE. Grey literature was also searched using the Google Scholar search engine. The searches included international peer-reviewed articles published in the period from 1996 (the year of the first GBD study [10, 14]) to 1 June 2016. The search terms were ‘cost’ OR ‘effectiveness’ OR ‘cost-effectiveness’ AND ‘disability adjusted life years’ AND ‘malaria’. Any literature which did not satisfy these criteria was excluded. The snowball technique was used to identify other articles by searching for relevant papers listed in reference lists of the initially selected articles. Although review papers were not included as part of this review, they used to check for other potential references that fulfil the eligibility criteria. A total of 82 studies were initially retrieved and after further screening using the inclusion criteria, a total of 40 studies were finally included for this review. The inclusion criteria are shown by the PRISMA flow diagram (Fig. 1) which was adapted from Moher et al. [16] and modified. We limited our search to papers written in English. For each of the selected studies, we noted the year of study, malaria intervention assessed, intervention target population, country of study, data sources and CERs in terms of DALYs averted. We checked if the studies followed the GBD study methodology in estimating DALYs. We assessed the use of disability weights, application of age weighting and discounting as well as use of life expectancy tables.

### Cost-effectiveness analysis

Reviewed studies used two main approaches as thresholds to determine whether or not an intervention was cost-effective. The first approach was based on per capita gross domestic product (GDP). Interventions with a CER per DALY averted less than a country’s per capita gross domestic product (GDP) could be regarded as ‘very cost-effective’ and those for which the cost-effectiveness is less than three times the country’s per capita GDP as ‘cost-effective’. This approach was recommended by the World Health Organisation’s choosing interventions that are cost-effective (WHO-CHOICE) project [15, 17]. The second approach used was thresholds of US$ 30 and US$ 150 per DALY averted as a basis for considering an intervention either highly cost-effective or cost-effective respectively [18, 19].

In order to standardise the CERs, each ratio was expressed as number of DALYs averted per US$ 1 million spent on the intervention. For CERs expressed as a range, the midpoint value was calculated and used calculate the number of DALYs averted.

## Results

### Characteristics of reviewed studies

**Fig. 2:**
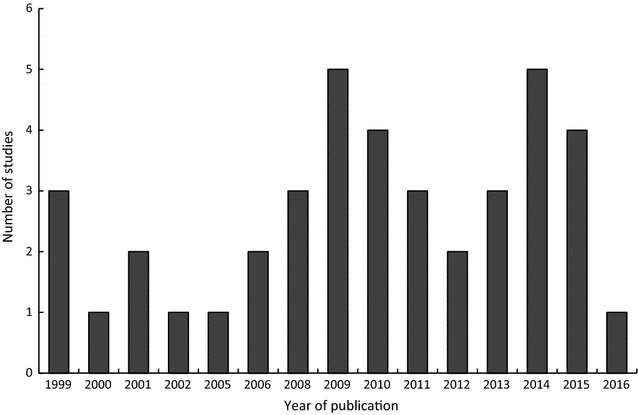
Number of reviewed studies per year (1999-June 2016) that used DALYs in cost-effectiveness analysis

Most of the reviewed studies (n = 30) were done in Africa or used the African settings to model the CEA. The studies were published between 1999 and 2016. Figure 2 shows the number of reviewed studies against the year of publication. The highest number of studies were published in 2009 and 2014. The studies were done using data at different levels of coverage; district (n = 11), provincial (n = 4), national (n = 3) and regional (n = 5) levels. Some studies (n = 17) did not specify the level of coverage as most of them used modelling to determine the cost-effectiveness of the interventions. Other studies (n = 21) stated the type of malaria (*Plasmodium falciparum* or *Plasmodium vivax* malaria) targeted by the intervention. Of the studies that stated the type of malaria, the majority of them were on interventions against *P. falciparum*. Sixty percent (60.0%) of the malaria interventions targeted all age categories, while the others targeted pregnant women (12.5%), children (25.0%) and both women and children (2.5%). Of the studies whose interventions targeted children, 20.0% (n = 8) of them specifically targeted infants.

### DALY methodology used

The DALY methodology was generally applied in a standard manner by most of the studies under review. However, some aspects of DALY calculation such disability weights and life expectancy values were not clearly stated in some instances. It was also not clear which specific assumptions were made when the calculation of DALYs was done. It was found that 19 studies (47.5%) applied the same disability weight for malaria from the global burden of disease (GBD) studies. The first GBD study was published in 1996 [10] and the latest one in 2015 [20]. Other studies either applied country or region specific disability weights. However, 15 studies (37.5%) did not clearly specify which disability weights for malaria were used to calculate the DALYs. Four (4) reviewed studies reported that they applied age weighting while six studies did not report on the use of age-weighting. Thirty-two (32) studies (82.5%) reported applying a 3% discounting rate while six studies did not clearly specify whether discounting was applied or not. Twenty-seven studies (67.5%) used country-specific life tables while 6 (15.0%) used life tables obtained from the GBD study or WHO. Some of the studies 6 (15.0%) did not explicitly state the source of the life expectancy used. The major sources of data used for calculating the DALYs included published and unpublished studies, malaria control program records, health facility records, clinical trials, programme reports and census data.

### Cost-effectiveness ratios

In this review, we identified 40 studies on CEA of malaria interventions, which used DALYs to determine CERs in terms of cost per DALY averted per each intervention. The CERs were expressed in United States Dollars (US$) per DALY averted and ranged from 1 US$ to 639 US$ depending with the specific intervention. Some studies expressed the CERs as cost of DALYs averted per person while in other studies it was expressed as the number of DALYs averted over a certain period of time. Other studies (15.0%) did not specify the exact number of DALYs averted per each intervention. To standardise the CERs, we also expressed cost-effectiveness as DALYs averted per 1 million US$ spent on each intervention. Based on internationally accepted thresholds for CERs [15, 17–19], most of the interventions (85%) were found to be cost-effective. For the malaria interventions that were targeted at all age groups (Table 1), pre-referral rectal antimalarial treatment and dihydroartemisinin–piperaquine (DP) combined with artemether–lumefantrine (AL) were the most cost-effective interventions. Each of these interventions averted approximately 200,000 DALYs per 1 million US$ spent on the intervention. In some cases, the same intervention was more cost-effective in one setting compared to a different setting. For example, intermittent preventive treatment had a CER of US$ 41.46 per DALY averted in Mozambique, while the same intervention had a CER of US$ 136.30 when it was carried out as part of a multi-country study.

**Table 1:** Summary of study characteristics and costs per DALY averted for interventions targeting all ages

References	Publication year	Country/region	Malaria intervention	Main data sources	Life tables source	Cost-effectiveness threshold used	Cost-effectiveness ratio (cost per DALY averted in US $)	DALYs averted per $ 1 million spent on intervention
[1]	1999	Brazil	Case treatment, vector control and insecticide spraying	Malaria control program	GBD study	Not stated	$69	14,493
[2]	1999	Model	ITNs	Model Assumptions	GBD study	US$ 30 and US$ 150	<$150	<6667
[3]	2001	Zambia	Environmental management, treatment and mosquito nets	Malaria control program data	West African model life table	Not stated	$524–591 over a period of 3–5 years	1799
[4]	2002	Zambia	Integrated malaria control (environmental management, rapid diagnosis and treatment and the use of bed nets	Census Hospital patient records	West African model life table	Not stated	$36.90 (average)	27,100
[5]	2005	Multi-country	Combination of strategies	Reports WHO databases Expert opinion Published data	WHO life tables	Not stated	$10–100	18,182
[6]	2006	Kenya	Malaria home management Educational programme	Census Published data Surveys	Model	US$ 30 and US$ 150	<$30	<33,333
[7]	2006	Non-specific	Case management	Simulation model	Ethiopian life table	Not stated	Not specified	
[8]	2008	Sub-Saharan	Diagnostic methods	Published and unpublished data Expert opinion	West African life table	US$ 150	>US$ 70 (95% certainty and $40% malaria prevalence)	>14,286
[9]	2008	Multi-country	IRS ITNs	Published data ITN and IRS programmes	Not specified	US$ 30 and US$ 150	$56 (calculated average)	17,857
[10]	2009	Kenya and Uganda	Early detection system	Health facilities		US$ 30 and US$ 150	<$150	<6667
[11]	2009	Non-specific	Vaccines (PEV, BSV, MSTBV and combinations)	Published data WHO estimates	Ethiopian life table	Not stated	$31 (pre-erythrocytic vaccine at $2 dose)	32,258
							$13.50 (blood stage vaccine at $2 dose)	74,074
[12]	2009	Eritrea	ITNs	Published data National malaria control programme	WHO	US$ 30 and US$ 150	$13–44	34,483
[13]	2010	Model	Pre-referral rectal antimalarial treatment	Published data World malaria report	Region-specific	US$ 30 and US$ 150	$5 (in SSA)	200,000
[14]	2011	Model	Pre-erythrocytic malaria Vaccine		Ethiopian life table	US$ 30 and US$ 150	I$207 (ceiling ratio)	4831
[15]	2012	Kenya	LLINs	Published data Epidemiologic data		Not stated	<$20	<50,000
[16]	2013	Experimental Model	LLINs	Published studies	Country specific	US$ 30 and US$ 150	US$ 235.28	4250
[17]	2013	Model	LLINs	Simulation	Country specific	US$ 30 and US$ 150	US$ 235.28	4250
[18]	2013	Multi-country	Pre-erythrocytic Vaccine	UN population division WHO	WHO	1–3 times GDP per capita	$56	17,857
[19]	2014	Tanzania	Larviciding	Malaria control program	GBD 2010	1-3 times GDP per capita	$ 43-$ 545 (depending on scenario)	3401
[20]	2014	Kenya	Combined interventions (IRS, LLINs, AL	Published data Survey	Ethiopian life table	Not stated	$4.29–$55.70 (depending on the intervention)	33,339
[21]	2015	Multi-country	Dihydroartemisinin–piperaquine (DP) Artemether–lumefantrine (AL)	Multi-centre clinical trial	WHO life table	Not stated	$ 5 (for DP)	200,000
[22]	2015	Myanmar	RDTs	Published data Management information system	World Bank Global burden of study (2012)	Not stated	$639^a^	1565
[23]	2015	China	Malaria elimination	Published data Infectious disease information system	Chinese life table	Not stated	Not specified	Not applicable
[24]	2016	Model	RTS,S/AS01 malaria vaccine		Ethiopian life table	Not stated	$ 80 (3 dose schedule at $5 per dose)	12,500

^a^Subsidy with information, education and counselling compared to no intervention

Combined interventions targeting pregnant women were the most cost-effective (Table 2). The combined interventions included provision of insecticide treated nets (ITNs), residual spraying, chemoprophylaxis and improved case management in pregnant women (IPTp) was the least cost-effective. The number of DALYs averted per 1 million US$ spent on each intervention ranged from 19,231 to 222,222 DALYs.

**Table 2:** Summary of study characteristics and costs per DALY averted for interventions targeting pregnant women

References	Publication year	Country/region	Malaria intervention	Main data sources	Life tables source	Cost-effectiveness threshold used	Cost-effectiveness ratio (cost per DALY averted)	DALYs averted per $ 1 million spent on intervention
[1]	1999	Sub-Saharan Africa	ITNs, residual spraying, chemoprophylaxis and improved malaria treatment	Published and unpublished sources Programmes	GBD study	US$ 30–US$ 150	$4–$10 for treatment of existing nets	142,857
							$19–$85 for residual spraying	19,231
							$3–$12 chemoprophylaxis for children	133,333
							$4–$29 for pregnant women treatment	58,997
							$1–$8 for case management	222,222
[2]	2001	Non-specific	Sulfadoxine–pyrimethamine	Published and unpublished data	Kenyan life table	Not stated	$10–14	83,333
[3]	2009	DRC	Insecticide treated nets (ITNs) distribution	Clinic records, program records and peer-reviewed literature	WHO life table	1–3 times GDP per capita	$17.22	58,072
[4]	2010	Mozambique	Intermittent preventive treatment	Demographic surveillance system	Mozambique life table	US$ 36–US$ 129	$41.46 (maternal malaria)	24,120
[5]	2015	Model	Intermittent preventive treatment	Published data	GBD (2010 and 2004)	1–3 times GDP per capita	$7.28	137,363
[6]	2015	Multi-country	Intermittent preventive treatment	Clinical trials Published data	Country-specific	US$ 240	$136.30	7337

Intermittent preventive treatment (IPT) in children (Table 3) in Mozambique and Tanzania was the most cost-effective intervention, averting 270,270 DALYs per 1 million US$ spent on the intervention. Vaccines and long lasting insecticide-treated nets (LLITNs) were the least cost effective interventions for children and infants.

**Table 3:** Summary of study characteristics and costs per DALY averted for interventions targeting children

References	Publication year	Country/region	Malaria intervention	Main data sources	Life tables source	Cost-effectiveness threshold used	Cost-effectiveness ratio (cost per DALY averted)	DALYs averted per $ 1 million spent on intervention
[1]	2000	Tanzania	Chemoprophylaxis	Clinical trial Passive case detection Survey	Model life table West 25 and 26	US$30–US$ 150	$11–12	86,956
[2]	2008	Togo	LLITNs	Regional estimates Financial records and interviews	Togo life tables	US$30–US$ 150	$16.39	61,013
[3]	2009	Mozambique and Tanzania	Intermittent preventive treatment in infants (IPTi)	Expanded program on immunization	East African life table	US$ 30 and US$ 150	$3.70 in Tanzania	270,270
							$11.20 in Mozambique	89,286
[4]	2010	Multi-country	Intermittent preventive Treatment (ITP)	Model	Country-specific	US$ 202	$2.90–$39.63	47,170
[5]	2010		Pre-referral rectal artenusate		Sub-Saharan Africa life table	1–3 times GDP per capita	I$77^c^ (at full uptake)	12,987
[6]	2011	Multi-country (Tanzania, Uganda, Nigeria)	Parenteral artenusate	Clinical trials	Country-specific (3 countries)	US$30–US$ 150	$123^a^	8130
[7]	2011	Model	Intermittent preventive treatment in infants (IPTi) and children (IPTc)	Simulation model Clinical trials	East African life table	US$ 37–US$223	<$100	<10,000
[8]	2014	Africa	Dihydroartemisinin–piperaquine (DP) and artemether–lumefantrine (AL)	Randomised trials	WHO African sub-region life table	Not stated	$0.96 per child over one year^b^	Not applicable
[9]	2014	Malawi	Vaccines and Lon lasting insecticide treated nets (LLITNs)	Published sources	Malawian life table	1–3 times GDP per capita	$145.03 (from a health service perspective	6895
[10]	2014	Tanzania	Dihydroartemisinin–piperaquine Artemether–lumefantrine	Published studies Health facility	Tanzanian life table	US$ 30–US$ 150 GDP per capita	$12.40–12.54	80,192

^a^These were DALYs averted after treatment with artenusate compared to quinine as a baseline

^b^First-line treatment with dihydroartemisinin–piperaquine (DP) compared to artemether–lumefantrine saved $0.96

^c^I$ stands for international dollars

The least cost-effective intervention was rapid diagnostic tests (RDTs) in Myanmar, averting only 1565 DALYs per 1 million US$ spent on the intervention. Although most interventions were cost-effective, some studies (35%) did not specify the specific thresholds that were applied to determine cost-effectiveness.

### Malaria interventions

Broad malaria intervention categories for the reviewed studies are summarised in Table 4. The interventions were classified as case treatment, prophylaxis, vector control, insecticide treated nets, early detection, environmental management, diagnosis, combined interventions and educational programmes. Most CEA studies were on case treatment (30.0%) and preventive treatment (30.0%) followed by insecticide treated bed-nets (27.5%). However, some studies analysed cost-effectiveness of more than one malaria intervention.

**Table 4:** Broad malaria intervention categories showing the number of studies as a percentage of the total number of reviewed studies

Intervention category	Number of studies (%)	References
Case treatment (out- and in-patients)	12 (30.0)	[3, 8, 21–30]
Prophylaxis	12 (30.0)	[3, 29, 31–40]
Vector control/insecticide spraying	6 (15.0)	[3, 27, 29, 30, 41, 42]
Insecticide treated nets	11 (27.5)	[3, 28–30, 40, 42–49]
Early detection system	1 (2.5)	[19]
Environmental management	2 (5.0)	[28, 50]
Diagnosis	2 (5.0)	[51, 52]
Educational programme	1 (2.5)	[53]
Malaria elimination program	1 (2.5)	[54]
Combination of interventions	6 (15.0)	[3, 27, 28, 30, 40, 50]

### Case treatment

Pre-referral artenusate was shown to be a cost-effective intervention in the management of severe childhood malaria in rural African settings with a cost of 77 international dollars (I$) per DALY averted at full uptake [25]. Parenteral artenusate was highly cost effective and an affordable alternative to quinine for treating children with severe malaria with a cost of $123 per DALY averted after treatment with artenusate compared to quinine as a baseline [8]. One study showed that combined rectal formulations (antimalarials and antibacterials) are a cost-effective alternative to rectal anti-malarials or anti-bacterials alone [22]. This intervention showed a cost of $5 per DALY averted. Three of the reviewed studies compared the cost-effectiveness of dihydroartemisinin–piperaquine (DP) and artemether–lumefantrine (AL) in treatment of complicated malaria [21, 23, 55]. The maximum cost per DALY averted for these three studies was $12.54. These two drugs are highly recommended for the treatment of uncomplicated malaria. It was predicted that DP was more cost-effective compared to AL with the assumption that compliance to treatment was higher in DP than in AL due to the once a day dose for DP [23]. It was also suggested that DP might be more cost effective than AL across a range of settings in Africa [55].

### Prophylaxis

Intermittent preventive treatment (IPT) is one of the malaria control strategies used in malaria endemic areas. This strategy is often used in infants and pregnant women and can contribute to a decrease in clinical malaria [32]. IPT in infants (IPTi) was shown to be a highly cost-effective intervention when it is delivered alongside the expanded programme on immunisation (EPI) with a range of $2.90–$29.63 per DALY averted [32]. This strategy can be strengthened by inclusion of iron supplementation [33]. IPTi with sulfadoxine pyrimethamine (SP) is expected to produce health improvements in a cost-effective way from both the health system and societal perspectives [31]. One study demonstrated the importance of CEA for IPT in pregnant women (IPTp) in supporting policymakers’ decisions [56]. The study showed that monthly doses of SP during the second and third trimester are more cost effective than only two doses that were previously recommended. This finding was consistent with the WHO guidelines [57].

One reviewed study estimated the cost-effectiveness of IPTp-SP on maternal clinical malaria and neonatal survival in Mozambique [38] and found that it was cost-effective in both instances. The cost per DALY averted for maternal malaria was $41.46. This intervention was said to remain cost-effective even with significant increases in drug and other related costs. Presumptive treatment regimens to prevent low birth weight associated with malaria were shown to be a cost-effective strategy in areas with high malaria transmission rates [26]. The cost-effectiveness of 2-dose IPTp-mefloquine (MQ) was compared with IPTp-SP in HIV negative women. IPTp-MQ was more cost-effective than IPTp-SP although poor tolerability of MQ does not favour its use for IPTp [58].

A malaria vaccine model was applied to analyse the cost-effectiveness of a hypothetical pre-erythrocytic malaria vaccine [34]. Using a ceiling ratio of I$207 as cost per DALY averted, this study showed that 52.4% of parameterizations predicted cost-effectiveness in the primary analysis. The cost-effectiveness of the vaccine was shown to be maximal in low endemicity settings, thereby suggesting the use of a selective vaccine introduction strategy. Vaccinating children with RTS,S vaccine was shown to be very cost-effective from both a societal and health service perspective in Malawi [40]. However, the study recommended that long-term follow-up studies were essential. The RTS,S/AS01 vaccine was shown to be highly cost-effective across a wide range of African settings [36].

Simulation showed that malaria vaccines might be an efficient malaria control intervention and that both transmission setting and vaccine delivery modality are important to their cost-effectiveness [39]. The simulation used three different vaccine types: pre-erythrocytic vaccines (PEV), blood stage vaccines (BSV) and mosquito-stage transmission-blocking vaccines (MSTBV). The cost per DALY averted for PEV and BSV was $31 and $13.50 respectively at a cost of $2 per dose. Specific malaria intervention drugs used in prophylaxis, case treatment and vaccines are summarised in Table 5. SP was the most common drug of choice in malaria prophylaxis, while artemisinin-based combination therapies (ACTs) were the most common drugs used in case treatment. In addition to prophylaxis, SP was also used for case treatment.

**Table 5:** Specific malaria intervention drugs used for prophylaxis, case treatment and vaccines

Prophylaxis	References	Case treatment	References	Vaccine	References
Sulfadoxine–pyrimethamine	[26, 29, 31, 32, 37, 38, 56, 58]	Dihydroartemisinin–piperaquine (DP)	[21, 23, 29, 55]	RTS,S	[34]
Mefloquine	[32, 58]	Artemether–lumefantrine (AL)	[21–23, 29, 30, 55]	Hypothetical	[35]
Chlorproguanil–dapsone	[32]	Artenusate	[8, 22, 25]	RTS,S/AS01	[36]
Artenusate	[32]	Quinine	[22]		
Amodiaquine–artenusate	[32, 37]	Sulfadoxine–pyrimethamine	[3, 24, 29]		
Pyrimethamine–dapsone	[33]	Amodiaquine	[24]		
Chloroquine	[3]	Chloroquine	[29]		

### Insecticide treated nets and vector control

The effectiveness of ITNs in preventing malaria is threatened by increasing resistance to insecticides as well as changing biting behaviour of mosquitoes [44, 45]. Combination mosquito nets such as pyrethroid piperonyl butoxide long lasting insecticidal nets were shown to be likely more cost effective than standard long lasting insecticidal nets (LLINs), especially in areas with strong resistance to pyrethroids [44]. Varying malaria transmission levels were shown to have an impact on CERs.

There is paucity of information on the cost-effectiveness of larviciding. In light of this, a study was carried out in Tanzania to estimate the CERs of microbial larviciding for malaria vectors [41]. The study estimated CERs from the provider and societal perspectives, and showed that larviciding can be used as a cost-effective intervention in urban areas with the cost per DALY averted in the range $43–$545. A study on the cost-effectiveness of a malaria control program showed case treatment to be more cost-effective than vector control, in particular, in areas where *P. falciparum* is prevalent and insecticide spraying is relatively ineffective [27]. The cost per DALY averted was shown to be $69.

The cost-effectiveness of elimination of falciparum malaria was analysed in a province in China [54], with the results of the study showing that the programme was cost-effective. The cost-effectiveness of an early detection system for epidemic malaria was explored in the highlands of Kenya and Uganda [19]. Results from the study suggested that the early detection system was cost-effective, but further studies are needed to analyse the costs and effects of the health systems’ reaction after being prompted by the early detection system.

There is scanty information on the cost-effectiveness of environmental management as a malaria control strategy. Hence, a study was conducted to assess the efficacy and cost-effectiveness of environmental management (vegetation clearance, modification of river boundaries, draining swamps, oil application to open water bodies and house screening) [28]. The results of that study showed that environmental management, when integrated with other malaria control interventions like case treatment, insecticide spraying and bed nets, could substantially increase the chances of rolling-back malaria. A study in rural Kenya found that an educational programme for home management of malaria targeted at shopkeepers and communities was highly cost-effective when compared to other benchmarks for interventions in resource-limited settings [53]. The strategy of introducing an education programme is therefore essential in areas where a large proportion of the community access malaria drugs from private retailers.

### Diagnosis

A subsidy of RDTs and artemisinin-based combination therapies (ACTs) within the informal private sector can help in the efforts to fight malaria. When these subsidies are combined with information, education and counselling, the results were shown to have favourable CERs [51]. RDTs were shown to have the potential to be cost-effective in most parts of sub-Saharan Africa. This reflected better treatment and health outcomes for non-malarial febrile illness as well as savings on antimalarial drug costs [52].

### Combination of interventions

A stochastic simulation modelling platform was applied to simulate the impact of interventions singly and in combination in the highlands of Kenya [30]. The study results showed that the greatest simulated health impact was from a combination of long-lasting insecticidal nets (LLIN) use by 80% of the population, 90% household coverage by IRS with deployment starting in April and intermittent screen and test of school children using AL with 80.0% coverage twice per term. It was also shown that high coverage with artemisinin-based combination treatments is the most cost-effective strategy in most countries in sub-Saharan Africa with the cost per DALY averted in the range $10–100 [29]. However, this alone is not enough if it is not combined with other interventions such as use of ITNs, IRS and ITP. The economic impact of malaria was assessed in the mining sector in the Zambian copperbelt. The study showed that integrated malaria control in the copper mining communities was a sound investment resulting in reduced direct malaria treatment costs and reduced indirect costs as a result of reduced work absenteeism [50].

## Discussion

We reviewed studies that utilised the DALY metric in cost-effectiveness analysis of malaria interventions. Although the reviewed studies used the DALY, there were some variations in methodology. Most of the interventions were within the WHO-CHOICE thresholds for cost-effectiveness. Some interventions were more cost-effective in one setting compared to a different setting. This shows that cost-effectiveness analysis may only be useful in the context of the choices available in a particular setting [59]. Although most interventions reviewed in this study were cost-effective based on set thresholds, the number of DALYs averted per one million US$ spent on each intervention were different. It is therefore essential for policymakers to compare results of cost-effectiveness analysis with as many relevant interventions as possible before making resource allocation decisions.

In general, a combination of interventions were more cost-effective than single interventions. Combined malaria interventions have been shown to deliver substantial efficiency gains compared to single interventions [48]. For instance, ITN distribution was shown to be a more cost-effective intervention when added to antenatal services [43]. It is essential to include long-term surveillance as part of ITN interventions, with particular attention to the age range over which rebound can occur [46]. It has been shown that emphasis on treatment as well as targeted vector control yielded significantly lower costs per life saved [27]. Although there is a wide range of cost-effective interventions available, provision of these intervention packages is often not affordable in low-income countries. There is therefore, need for external donors to assist with funding where possible [3]. Information from the studies in this review can be used to make decisions on which interventions can be effectively applied independently and which ones are mutually exclusive [4].

The majority of published studies on CEA of malaria interventions using DALYs focused on case treatment, use of insecticide treated nets and prophylaxis. This review showed that there is paucity of information on cost-effectiveness of other interventions such as early detection, environmental management, diagnostic services and educational programmes. Lack of cost-effectiveness information on some interventions makes it difficult to conduct a comprehensive comparison in order to guide policy-makers in decision-making. There is therefore need for more CEA studies on less explored malaria interventions to inform policy and to improve effectiveness of these interventions. The evidence provided by such studies will assist in guiding decisions at various levels [6]. From our literature search, there were no studies on cost-effectiveness of malaria interventions for a malaria outbreak scenario. It would be interesting to know whether interventions that are likely to be cost-effective in a normal malaria transmission situation will also be cost-effective in an outbreak.

This review showed that disease modelling methods can provide useful information by predicting cost-effectiveness for scenarios and multiple strategies, where, for practical reasons, trials cannot be carried out [3, 37]. However, CEA results obtained through modelling techniques must be interpreted with caution as assumptions made in the models may be different to the actual situation obtaining in real life situations. Thus, results from complex models should be presented to decision-makers in a form in which interpretation and translation is easy.

Comparison of CERs among the reviewed studies was difficult as the number of DALYs averted per each intervention were often expressed differently. Although most studies expressed CERs as cost per DALYs averted, some studies only gave a range without specifying the mean number of DALYs averted. Some of the studies looking at a combination of interventions did not give a breakdown of the cost per DALY averted for each individual intervention. In some cases, there was very little information on the methodological choices made. For example, some studies did not specify the disability weight which was used, the sources of data on malaria incidence and the source of life expectancy values. In some cases, this information had to be extracted from referenced articles as it was not clearly stated.

## Conclusions

Cost-effectiveness analysis studies of malaria interventions done over the past 20 or so years have provided important information for policymakers to guide them on choosing the most cost-effective interventions for malaria programmes. This review has shown that most malaria interventions are cost-effective in terms of the cost per DALYs averted per each malaria intervention, based on acceptable thresholds. This information is useful in identifying interventions that effectively use available resources. Although most of the studies we reviewed generally followed the DALY methodology in the CEA, there were differences in the way the CERs were expressed thereby making it difficult to make a comprehensive comparison between studies.

## Abbreviations

AL: artemether–lumefantrine
CEA: cost-effectiveness analysis
CER: cost-effectiveness ratio
DP: dihydroartemisinin–piperaquine
DALY: disability adjusted life year
GBD: global burden of disease
GDP: gross domestic product
ITP: intermittent preventive treatment
ITNs: insecticidal treated nets
RDT: rapid diagnostic test
US$: United States Dollars
WHO: World Health Organisation
